# Protein-Losing Enteropathy in the Setting of Severe Iron Deficiency Anemia

**DOI:** 10.1177/2324709618760078

**Published:** 2018-02-26

**Authors:** Jessica Lacy Yasuda, Paul A. Rufo

**Affiliations:** 1 Boston Children’s Hospital, Boston, MA, USA

**Keywords:** protein-losing enteropathy, PLE, whole cow milk, iron deficiency, anemia, anasarca

## Abstract

A 20-month-old boy presented with a 2-week history of pallor and progressive abdominal distention. Nutritional history revealed long-standing excessive cow milk intake. He was subsequently found to be profoundly iron deficient and hypoproteinemic, with an elevated fecal α-1-antitrypsin level and occult blood positive stool, consistent with protein-losing enteropathy. He was treated with cow milk restriction and oral iron supplements, which resulted in resolution of his edema and laboratory anomalies. While small numbers of previous case reports have described the potential association between excessive cow milk intake and severe iron deficiency and protein-losing enteropathy, this constellation of clinical symptoms is infrequently recognized in clinical practice. As iron deficiency is recognized as the most common nutritional deficiency in the United States, it is important to keep excessive cow milk intake in mind when evaluating young children presenting with severe iron deficiency and protein-losing enteropathy.

## Introduction

Protein-losing enteropathy (PLE) occurs as a result of diverse anatomic, inflammatory, allergic, and infectious disorders that result in a common clinical presentation characterized as the excessive loss of serum proteins into the gastrointestinal tract. The differential diagnosis for PLE is divided into erosive processes affecting the gastrointestinal mucosa (as in inflammatory bowel disease), nonerosive gastrointestinal processes (as in celiac disease), and disorders of increased lymphatic pressure or increased gastrointestinal lymphatic losses (as in right heart failure or obstruction of the thoracic duct).^1^ However, some presentations of PLE defy placement in a discrete category, and this includes relatively rare cases of significant PLE and hypoalbuminemia observed in toddlers and young children presenting with severe iron deficiency anemia in the context of excessive cow milk intake.^2-8^ The majority of the published cases of infants and children presenting with severe iron deficiency anemia and PLE date back to the 1950s and 1960s^2-4^ and predate the widespread practice of iron fortification of cereals and formulas.^9^ Since then, only sporadic reports of children with severe iron deficiency anemia and PLE have been published.^5-8^ Here, we describe a case of PLE in a toddler who was found to have significant iron deficiency anemia in the setting of excessive cow milk intake.

## Case Description

A previously healthy 20-month-old boy presented for evaluation of progressive generalized swelling. His parents had first noticed a mild periorbital edema 2 weeks prior, and they reported that the swelling had progressed steadily over time, eventually resulting in worsening abdominal distension and bilateral lower extremity edema. Clinical history obtained in the office revealed that shortly after the edema was noted, the patient began passing up to 5 to 6 loose to watery, grossly nonbloody stools per day. Additional history revealed that the boy routinely drank more than 28 ounces of cow milk per day. Laboratory studies obtained at his pediatrician’s office revealed an albumin of 1.8 g/dL, and a urinalysis that was negative for protein. The patient was referred for further evaluation.

Laboratory studies obtained in the emergency department were consistent with a microcytic anemia (hemoglobin of 6.4 mg/dL with mean corpuscular volume of 69 fL), iron deficiency (ferritin of 4.9 ng/L), and hypoproteinemia (albumin of 1.6 g/dL, total protein of 3.0 g/dL). His fecal α-1-antitrypsin level was markedly elevated beyond the quantifiable upper limit of our testing (>1.13 mg/g), and his stool tested positive for occult blood. Workup included performance of upper endoscopy and colonoscopy, and these studies revealed grossly normal appearing mucosa lining the upper and lower gastrointestinal tract. Subsequent histologic examination revealed only a mild increase in the number of eosinophils in the gastric antrum and otherwise normal-appearing mucosa without the presence of acute or chronic inflammation in his esophagus, duodenum, colon, and rectum.

The patient received 1 transfusion (20 mL/kg) of packed red blood cells and was treated with oral iron replacement at a dose of 6 mg/kg of elemental iron/day. Cow milk was empirically eliminated from his diet, and he was instead placed on an amino acid–based formula as an intervention to address the possibility of an unusually late presentation of cow milk protein intolerance. He responded favorably to this combination of therapy. He has had no subsequent episodes of edema and his diarrhea resolved.

His albumin normalized within 1 week and his edema resolved completely. After 3 months of oral iron therapy, his hemoglobin had risen to 11.8 g/dL and his ferritin was 29.4 ng/L.

## Discussion

The American Academy of Pediatrics recommends screening all children at 12 months of age for iron deficiency anemia.^10^ Iron deficiency is the most common nutritional deficiency and affects up to 3% of children in the United States aged between 1 and 3 years.^10-12^ However, the occurrence of PLE in the context of iron deficiency anemia and excessive cow milk intake in a toddler or young child is a considerably rarer occurence.^2-8^ The typical patient presenting with this clinical picture is a toddler whose long-standing cow milk intake often exceeds from 24 to 30 ounces per day. Stool quality is inconsistently described in previous reports, and affected children can manifest stooling patterns that range from no change from baseline, to diarrhea,^2,3^ to diarrhea alternating with constipation.^8^ These children can develop progressive and generalized edema over a span of as little as 2 to 3 weeks. Laboratory testing confirms iron deficiency and severe microcytic anemia, hypoproteinemia, and PLE.^5,6,8^ Treatment with oral iron corrects the hypoproteinemia and clinical edema within 1 to 3 weeks. The underlying iron deficiency anemia resolves more slowly and typically over the course of months.^5,6,8^

Occult fecal blood loss has been described in children presenting with iron deficiency anemia–associated PLE, though the site of gastrointestinal blood loss is unclear. Six of 8 children described in a Finnish case series of iron deficiency anemia–associated PLE had positive stool occult blood testing.^6^ Upper endoscopic findings in these 8 Finnish children with PLE and iron deficiency did not support an erosive or inflammatory process in the stomach or duodenum as the etiology of their microscopic bleeding and iron deficiency–associated PLE. Jejunal biopsies in the affected children compared with 13 age-matched, healthy controls only showed minimal changes in mucosal structure with slightly elongated crypts, reduction in intraepithelial lymphocytes, and depletion of immunoglobulin A–containing cells.^6^ Jejunal findings in one toddler described in a separate case report included normal crypt architecture but increased eosinophils in the lamina propria.^5^ Due to the technical limitations of endoscopy, it is unclear if these children or the patient described in this report had mucosal injury in the distal jejunum or ileum.

We were unable to find any data from case reports of children with iron deficiency and PLE who underwent colonoscopy. Existing histopathological findings may suggest that there are distinct mechanisms that differentiate iron deficiency–associated PLE and cow milk protein intolerance from the more classic patient that presents with macroscopic erosive colitis, nodular lymphoid hyperplasia, and increased duodenal and rectal tissue eosinophilia.^13-15^ However, more colonoscopic studies in patients with iron deficiency–associated PLE will be necessary before we can fully characterize the differences between these 2 entities. The patient in this report had both visually and histopathologically normally appearing mucosa at the time of his colonoscopic studies, pointing away from primary large bowel inflammation as the origin of his occult fecal blood loss.

While allergy or hypersensitivity to cow’s milk protein has been hypothesized to be the primary precipitant for the PLE observed in these patients with severe iron deficiency, data from the available literature to date do not uniformly support this notion. In a small case series of Finnish children with PLE in the setting of excessive cow milk intake and iron deficiency anemia, treatment with oral iron corrected the laboratory abnormalities and clinical signs in these patients regardless of whether cow milk intake was eliminated.^6^ This clinical outcome was reproducible in another study that randomized 24 children with iron deficiency anemia (7 of whom had PLE) to receive either 16 ounces per day of whole cow’s milk or soy milk. Both groups were treated with 6 mg/kg/day of elemental iron. Interestingly, patients in this study experienced a complete resolution of PLE, in the presence or absence of ongoing enteral cow or soy milk protein exposure.^7^ As such, these data have led some investigators to conclude that intolerance to cow milk itself may not be the primary driver of the PLE in these patients, and instead iron deficiency may itself play a causative role.^5,6^ The rapid response of hypoproteinemia and edema to the introduction of iron therapy has been hypothesized by some investigators as support for iron deficiency as the driver of gut dysfunction leading to PLE.^6^

The gravity of the long-term neurocognitive effects of iron deficiency is becoming increasingly well understood.^10^ However, the effects of iron deficiency on other organ systems has been less well characterized. Iron is a critical nutrient that is incorporated into many metabolically noteworthy enzymes including hemoglobin (necessary for oxygen transport), components of the mitochondrial electron transport chain (important for ATP generation), enzymes critical for DNA synthesis, as well as the ubiquitous cytochrome P450 enzymes that are critical for detoxification and metabolite synthesis.^16^ The mechanism(s) that explain how iron deficiency can result in PLE remain unknown. That being said, it is clear that severe iron deficiency (and subsequent anemia) is very likely to affect adversely on tissue metabolism in the mucosa lining the small and large intestine, as well as other important and metabolically active tissues and organs throughout the body. In otherwise healthy individuals, most of the plasma proteins that pass into the gastrointestinal tract are degraded into amino acids by gastric and pancreatic proteases and subsequently reabsorbed by brush border transporters.^17^ Severe iron deficiency could lead to deranged function of the gut epithelial barrier, or may interrupt the production or function of peptidases and amino acid transporters important in protein recirculation. As such, iron deficiency/anemia may contribute to intestinal barrier dysfunction that results in either an increased pathologic transit of plasma proteins into the stool or decreased reabsorption of necessary amino acids from the intestinal lumen. It seems that a certain threshold of iron deficiency severity must be crossed for the PLE phenotype to develop, as most children with iron deficiency anemia do not present with clinically apparent edema. Additional studies are needed to clarify the role of iron in maintaining barrier function and protein-handling in the gastrointestinal tract.

Here we present a case of gastrointestinal dysfunction manifested as PLE in the setting of severe iron deficiency and excessive cow milk intake. This case highlights the importance of taking a thorough nutritional history in toddlers being assessed for iron deficiency anemia. Severe cases of iron deficiency anemia have been associated with PLE and may respond to supplemental iron therapy and/or cow milk protein restriction.
